# Drug waste minimization as an effective strategy of cost-containment in Oncology

**DOI:** 10.1186/1472-6963-14-57

**Published:** 2014-02-07

**Authors:** Gianpiero Fasola, Giuseppe Aprile, Luisa Marini, Alessandro Follador, Mauro Mansutti, Manuela Miscoria

**Affiliations:** 1 Oncology Department, University Hospital of Udine, 33100 Udine, Italy; 2 Pharmacy Department, University Hospital of Udine, Udine 33100, Italy; 3 Oncology Department, University Hospital of Trieste, 34100 Trieste, Italy

**Keywords:** Cost-containment, Oncology, Drug waste

## Abstract

**Background:**

Sustainability of cancer care is a crucial issue for health care systems worldwide, even more during a time of economic recession. Low-cost measures are highly desirable to contain and reduce expenditures without impairing the quality of care. In this paper we aim to demonstrate the efficacy of drug waste minimization in reducing drug-related costs and its importance as a structural measure in health care management.

**Methods:**

We first recorded intravenous cancer drugs prescription and amount of drug waste at the Oncology Department of Udine, Italy. Than we developed and applied a protocol for drug waste minimization based on per-pathology/per-drug scheduling of chemotherapies and pre-planned rounding of dosages.

**Results:**

Before the protocol, drug wastage accounted for 8,3% of the Department annual drug expenditure. Over 70% of these costs were attributable to six drugs (cetuximab, docetaxel, gemcitabine, oxaliplatin, pemetrexed and trastuzumab) that we named ‘hot drugs’. Since the protocol introduction, we observed a 45% reduction in the drug waste expenditure. This benefit was confirmed in the following years and drug waste minimazion was able to limit the impact of new pricely drugs on the Department expenditures.

**Conclusions:**

Facing current budgetary constraints, the application of a drug waste minimization model is effective in drug cost containment and may produce durable benefits.

## Background

The burden of cancer is increasing, producing skyrocketing costs which have a significant impact on health care expenditure in all developed countries. Urgent solutions to contain those costs are necessary and range from re-engineering the macroeconomic basis of cancer spending (e.g. value-based approaches to bend the cost curve and allow cost saving technologies), educating policy makers, introducing transparent and equitable regulatory systems, and adopting validated outcomes in both clinical trials and processes of drug approval [1,2].

The ASCO statement on the spiralling costs of cancer care suggest that the multiple factors lying beneath the rising expenditures in Oncology are a consequence of insufficient integration and coordination in health care systems [3]. Among possible solutions, measures to help oncologists to use cost-effectiveness information and discuss drug costs with their patients have been advocated [4].

In 2007 cancer costs accounted for around 6% of health care costs in Europe [5]. Total attributable expenses of cancer care include direct costs, such as drug expenditure, and indirect costs, mainly loss of productivity in individuals of working age [6].

Although prescription drug expenditure is only a small percentage of health care costs [6], antineoplastics alone represent a significant and growing part of this spending (~15% [5]) and are the leading category in hospital drugs expenses [7]. Even though few data are available, drug-related expenditure is proportionally higher in Oncology than in other medical specialities and overcome staffing costs for outpatients care [8,9].

Between 2005 and 2006 a 20% increase in cancer drug costs was observed in the U.S., mainly caused by the introduction of novel targeted therapies, such as bevacizumab, cetuximab and trastuzumab [10]. Similar reports have been produced in other Countries [11]. Furthermore, in the last 4 years, over 90% of the new cancer drugs approved by the FDA exceeded $ 20,000 for 12 weeks of treatment [12], rising sparkling debates on their cost-effectiveness [13].

During an economic recession, the rising costs of cancer treatment make therapeutic recommendations and cancer care management more complex. However, this is not a recession-specific issue, and middle income countries that are establishing universal drug coverage programs are also dealing with this dilemma [14,15]. European countries have faced the problem in many different ways: - through a government direct price control (Greece, France, Spain); - through a risk sharing policy (Italy); - through a ‘value-based’ pricing scheme (UK, Germany) [16]. In this scenario, the development of additional, low-cost measures to contain and reduce expenditures, such as drug waste reduction and human resources optimization, are highly desirable. Drug waste consists of the either unavoidable or inappropriate clearance of partially used ampoules, vials, syringes of drugs [17]. We have previously shown that a protocol including a centralized drug use surveillance combined with a intravenous (iv) chemotherapies scheduling by tumour type on different days and a planned dose rounding (up to 5% of the calculated dose [18]) is able to provide significant decreases in drug waste costs within the first year of application [19]. In 2005 at the Oncology Department of the University Hospital of Udine, the cost of iv consumed drugs reached 2,147,169 € with 8.3% of this expenditure (corresponding to 179,576 €) being attributable to drug waste. About 74% of all drug waste costs (133,292 €), corresponding to 6.2% of the Department drug costs, was attributable to six compounds (hereafter defined as ‘hot drugs’): cetuximab, docetaxel, gemcitabine, oxaliplatin, pemetrexed and trastuzumab. After the introduction of specific corrective measures [20], the total waste costs progressively dropped down, cutting by half the annual cost of waste. Still, over 80% of the waste expenditure was due to the six ‘hot drugs’. However, their waste decreased significantly (around 68%) leading to a reduction in the overall cost of waste, that was reduced to 4% of the global drug expenditure at that point [20].

In the present study we aimed to:

1. verify if the model may endure for a longer time, and confirm if the results we have obtained after the first year of application may further hold up

2. test if the virtual loop created by the application of the model may absorb an external disturbance. This was obtained with the evaluation of the impact produced by the introduction of new high-cost IV drugs in the clinical practice;

3. analyze potential pitfalls and propose new strategies to improve the final outcomes.

## Methods

The Department of Oncology at the University Hospital of Udine is a fully computerized, research-oriented clinical unit with approximately 1,500 new cases per year. The Department annual drug expenditure, defined as the sum of iv drug, including waste, plus oral cancer agents plus residual drug deposit) was 6,178,000 € in 2011. Drug dilutions for the whole Hospital are carried out at a centralized Antiblastic Drugs Unit (ADU). All prescribed chemotherapies are recorded in the computer system. This way pharmacists and technicians double check and record all the prescriptions (drug doses, dose rounding and reductions) at the ADU. The average number of diluted cycles per month for the whole hospital is over 1,300, with an average of 800 cycles per month (range in the years 2007-2009: 763-821) for the Oncology Department only.

Starting 2005, the number of monthly dilutions of all the iv drugs, day prescriptions and actual consumption have been recorded, calculating the actual use and the waste. The projected waste cost for the year and its proportion, compared to the overall pharmaceutical expenditure, have been calculated for the most expensive drugs.

After a period of observation, in January 2006 we introduced a protocol of waste reduction, which consists of four corrective measures:

1. a per pathology/per drug distribution of chemotherapy sessions over the week;

2. the choice of multi-dose vials, able to maintain micro-biological and chemical stability for up to 24 hours;

3. the rounding of drug dosages within 5% of the calculated dose [18,21]

4. the selection of the most convenient vial size, according to the drug unit price and to an accurate estimate of the daily use of each drug.

Starting July 2006, observation was focused on the six drugs which had shown to have the largest impact in terms of drug waste costs (cetuximab, docetaxel, gemcitabine, oxaliplatin, pemetrexed and trastuzumab), accounting for 88% of the waste costs and 3.5% of the Department annual drug costs. Those compounds were defined as ‘hot drugs’ since they had the highest impact on our annual budget due to both high unitary cost per milligram and increasingly high volumes of utilization. After approval in clinical practice and in the light of the high cost and the predicted increased prescription, we included bevacizumab (from 2007) and panitumumab (from 2009) in our analysis. Thus a total of 32 different drugs were considered: waste related to the 8 ‘hot drugs’ was registered monthly, while the one derived from the other 24 drugs on a six-month bases. Any decrease in negotiated drug prices occurred during the observation was taken into account in the economic analysis when comparing drug waste costs along the year.

In the current study we present the results of the 3 years follow up.

## Results

We present the results of the cost-containment policy study split into years. Average figures of monthly dilutions were consistent during the years of observation. The Department drug costs, observed iv drug costs and waste costs are synthesized in Table 1.

**Table 1:** Costs during the protocol application

	***2005***	***2006***	***2007***	***2008***	***2009***
***iv drugs***	2,147.169€	2,109.392€	3,062.369€	3,383.658€	3,471.665€
***Whole waste***	179,576€ (8,3%)	85,982€ (4%)	73,975€ (2,4%)	75,909€ (2,2%)	62,994€ (1,8%)
***Hot drugs waste***	133,292€ (6,2%)	75,788€ (3,5%)	51,441€ (1,6%)	53,386€ (1,5%)	54,721€ (1,5%)

### What happened in 2007

After the introduction of bevacizumab among the monitored compounds, their total waste costs was € 51,441, with an average per month of € 4,287. For the other 24 drugs included in the analysis, average drug waste cost per month was € 1,877. In just one year (2007) € 24,347 were saved from the waste of the monitored ‘hot drugs’, corresponding to a 32% cost reduction. Notably, 71% of the total waste expenditures may be attributed to pemetrexed and trastuzumab. Cost of waste for bevacizumab represented 7.9% of the expenditures of the ‘hot drugs’ (€ 4,056).

### What happened in 2008

During 2008, the total waste cost for the ‘hot drugs’ were consistent with 2007, being of € 53,386. In 2008, total savings amounted to € 22,402, corresponding to a percentage reduction of drug waste cost of 29%. Once again, 87% of the waste expenditures came from pemetrexed and trastuzumab. Notably, whilst we observed an increase in prescriptions and dilutions of pemetrexed (+39.5%), docetaxel (+14%), gemcitabine (+9.5%), oxaliplatin (+21.7%) and bevacizumab (+61%), the overall drug waste expenditures for the seven ‘hot drugs’ remained comparable to that of 2007. This can be partially justified by a concurrent decrease of cetuximab (-10%) and trastuzumab (-11.6%) prescriptions. In 2008 cetuximab waste accounted for 3.5% (€ 2,680) and trastuzumab waste for 18.8% (€ 14,283) of the total cost of waste.

### What happened in 2009

The introduction of panitumumab in our analysis (2009) was linked to a total waste expenditure for this drug of € 1,046 (2%).

During 2009 total cost savings for the eight ‘hot drugs’ were consistent with the previous years (€ 21,067 corresponding to a 27.8% reduction on 2006 costs).

**Figure 1:**
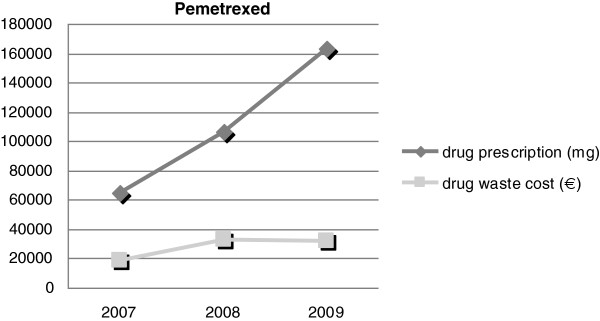
Pemetrexed waste cost compared to its prescription.

**Figure 2:**
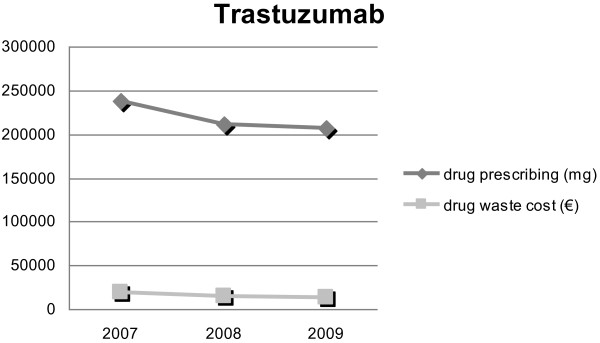
Trastuzumab waste cost compared to its prescription.

Compared to 2008, in 2009 we recorded a significant increase in prescriptions of pemetrexed (+34.8%), cetuximab (+35%), oxaliplatin (+44.5%) and bevacizumab (+60%). Pemetrexed and trastuzumab were responsible themselves for 50.6% (€ 31,901) and 21.7% (€ 13,707) of the total waste costs Figures 1 and 2.

In 2009 the negotiated price of oxaliplatin decreased sensibly, due to patent expiry. Thus we analysed the waste percentage per month and the consequent cost of waste in 2008 and 2009. During 2008 the waste cost of oxaliplatin accounted for 0.8% of the whole expenditures (€ 421). After the price reduction, this cost decreased in 2009 to € 110 (0.2%). However, despite an increase in the prescription of oxaliplatin of 44% compared to 2008, even if the price per milligram had remained the same as in the previous year, in 2009 the total cost for oxaliplatin waste would have accounted only for 1.5% of the total expenditure (€ 807).

## Discussion

### How a simple cost-saving policy may fit in the current landscape

The rising cost of cancer care has progressively become hardly sustainable [22]. Whether will be possible to contain health care costs while maintaining open access to cure and without worsening the results of cancer care is uncertain [2,20]. The continuing progress in oncology has to face an increasing need of rationalization of expenses. Many of the strategies proposed to reduce health care cost are medium to long term possible solutions whose impact on expenditures is barely predictable [23]. Low cost measures with a rapid effect on spending containment and able to optimize human and economic resources are highly desirable.

We have previously shown how a simple policy of drug waste control may significantly decrease the impact on the overall pharmaceutical expenditures and allow a substantial cost saving in the short time. In the present study, we also demonstrate that these measures can provide a long lasting benefit, although slightly diluted with time. Moreover, the application of the proposed model can reduce the impact of the waste of expensive drugs on the rise of costs.

Notably, monitoring the 24 drugs with low impact on cost of waste, we observed that waste expenditure was less than € 2,000 per month. Thus, with the protocol application, the waste for the non-hot drugs accounted for around 2-3% of their global cost. This percentage can be considered as the most favourable result from the protocol application and could be adopted as a benchmark to compare the efficiency of the model in waste costs containment.

**Figure 3:**
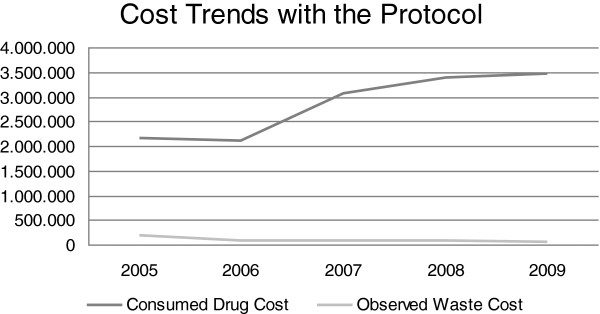
Comparison between iv Drug Costs and Waste Costs after the application of the protocol.

**Figure 4:**
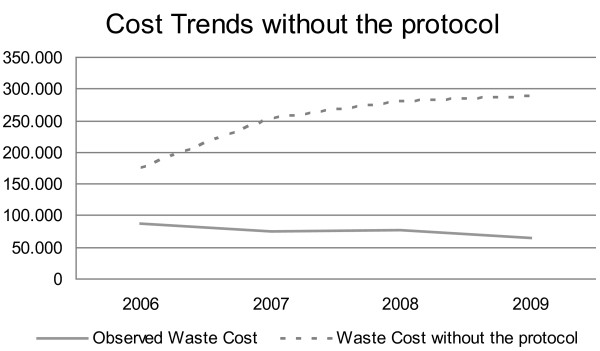
Waste Costs observed and estimated without the protocol adoption.

**Figure 5:**
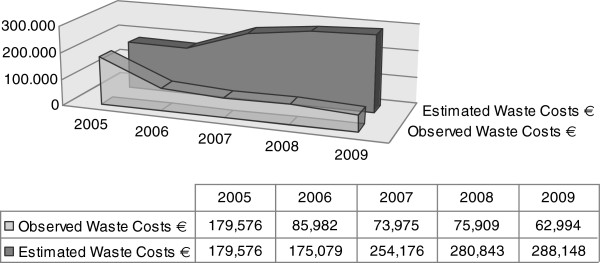
Comparison between Observed Waste Costs and Estimated Waste Costs without the protocol.

In 2005 drug wastage accounted for 8.3% of the Department annual drug expenditure, corresponding to € 179,576. If the same percentage of drug wasting had been confirmed in the following years, the cost of wastage would have accounted for € 175,079 in 2007, € 280,243 in 2008 and € 288,248 in 2009 Table 2; Figures 3, 4 and 5.

**Table 2:** Calculated savings per year

	**2006**	**2007**	**2008**	**2009**
***Waste***				
*Estimated*	175,079€	254,176€	280,843€	288,148€
*Observed*	85,982€	73,975€	75,909€	62,994€
***Saving***	**89,097€**	**180,201€**	**204,934€**	**225,154€**
***Hot drugs waste***				
*Estimated*	130,782€	189,866€	209,786€	215,243€
*Observed*	75,788€	51,441	53,385€	54,721€
***Saving***	**54,994€**	**138,425€**	**156,400€**	**160,522€**

(Estimated = calculated on 2005 waste percentage; Observed = with drug waste minimization).

Most waste costs were due to six ‘hot drugs’ (cetuximab, docetaxel, gemcitabine, oxaliplatin, pemetrexed and trastuzumab). After the introduction of waste limiting measures, in 2006 we observed a meaningful reduction (45%) in drug waste expenditure. This benefit was confirmed during the following 3 years. In fact, considering the ‘hot drugs’ waste, the application of our protocol produced a saving of € 24,347 in 2007, € 22,403 in 2008 and € 21,067 in 2009 (that is 32%, 29% and 27% of the iv drugs waste costs) in addiction to over € 90,000 per annum saved since 2006 (when comparing to the pre-protocol era) even after the introduction of new high cost drugs.

After the inclusion in our observation of panitumumab (2009), despite the high-cost per mg (4.2 €/mg in Italy), within the protocol its prescription did not cause any increase in drug waste expenditures (accounting for 1.6%).

These results suggest that not only the price per milligram (mg) is a critical element in drug waste control, but also the mgs wasted themselves. This is further supported by the trend in the cost of waste of oxaliplatin. In this case the price per mg substantially decreased in 2009 and the total amount of its waste cost declined consistently with price reduction. However, compared to the total waste costs, oxaliplatin waste accounted for a very small percentage (less than € 1,000 per month).

Furthermore, despite the increased prescriptions of several drugs (pemetrexed, docetaxel, gemcitabine, oxaliplatin, bevacizumab), the proportion of waste did not change. This evidence supports the efficacy of our protocol in containing drug wastage and reducing its costs, independently of the amount of drug prescribed.

During the 3 years of observation, the greatest amount of waste expenditures came from pemetrexed and trastuzumab. Thus, according to our experience, after a period of waste recording and observation, the monitoring of these two drugs is effective in estimating overall drug wastage.

In particular, pemetrexed prescriptions have increased during 2008 as a result of the approval of metastatic NSCLC in the first line treatment by the Italian Drugs Monitoring Controlling Body (AIFA). In light of the observation that wasted mgs have a decisive influence on the total waste expenditure per drug, the high rate of pemetrexed wastage can be attributable to the dose per ampoule which did not allow a favourable rounding. Starting 2010, pemetrexed has been available in 100 mg ampoules, that has facilitated an accurate dosing and has further reduced waste. If it is confirmed that ampoules dosages have a strong influence in drugs wastage, in future at the time of negotiation with the Drug Companies, attention should be paid not only to price per mg, but also to mg per ampoule, drug stability and vials.

Our protocol on drug waste minimization has proved to be effective in drug costs containment and to be consistent and with durable results. It would be thus interesting to verify the reproducibility of these results in other hospitals. In fact, the observation ended in 2009 after almost 4 years of follow up. This experience was extended to a large number of prescriptions and dilutions and the results we observed in 2007, 2008 and 2009 were reliable in terms of drug waste savings.

### Limitations of the study

Limitations of our study include that it is a monocentric experience, carried out in a fully computerized clinical unit. In our point of view, an adequate computer system and a centralized ADU are required for the application of the protocol, together with an adequate training of the personnel involved. Secondly, we did not considered the impact of oral high-cost drugs on drug expenditures, which may need a specific protocol to evaluate the amount of drug waste and related costs and ad-hoc measures to reduce them. Moreover we have to consider that, in the near future, the fraction of iv drugs will proportionally decrease, and the availability of biosimilar cancer drugs will also help reducing the costs [24,25] Finally, after a significant decrease in wastage costs during the first year of application of the protocol, the benefit tends to reach a plateau, with smaller savings during the second and third year. On the other hand this can be positively interpreted as the ability of the system to maintain its steady state after the first phase of optimization, supporting the robustness of our measures with time.

### Future developments and possible scenarios

Possible future developments of our protocol include the evaluation of the efficacy of dose rounding other than 5% of the whole dose. In fact, whilst this rounding is validated for chemotherapy agents [21], and it has been recently suggested that biologic agents rounding would be the same [26]. Recent data suggest that up to 10% rounding can be effective, with no detrimental effect on drug efficacy [27]. Based on preclinical and early clinical data on the anticancer activity of biologic products [28,29], Winger et al. showed how dose rounding to a value within 10% of the ordered doses could produce a 42% reduction in drug wastage. Effective drug-target interactions, neutralizing antibody formation and biologic characteristics of the disease impact on drug activity more than drug dosage itself. Thus, the amount of administered drug should be adequate to yield systemic concentrations that will optimally saturate or modulate the target. The application of these observations to our protocol might further increase drug waste savings from iv high-cost drugs. Moreover, a similar approaching strategy may be applied to oral cancer agents (both chemotherapies and biologics) to save remnants and further reduce healthcare costs. In conclusion, the present study demonstrates that drug waste surveillance is a low-cost, effective and lasting measure to substantially reduce intravenous antineoplastic expenditures.

## Conclusions

We have shown how the application of a simple drug waste minimization model may consent to cut by half the whole drug waste expenditures. Facing current budgetary constraints, this policy was effective in cost containment and produced long-lasting benefits. Our experience represents a contribution towards the sustainability of the costs of cancer care and its value in cost-containment could be proven and better quantified through multicentric experiences on larger areas. Among major suggested changes of the oncologists’ behaviour in attitudes and practice [20], we think that low-cost measures producing a more responsible drug prescription may be highly attractive.

## Competing interests

All authors (GF, GA, LM, AF, MM, MM) have no financial or non-financial competing interests to declare or disclose.

## Authors’ contributions

GF, GA, and MM: study conception and design. LM, MM: data acquisition. GF, GA, LM, AF, MM, MM: data analysis and data interpretation. GF, GA, MM: manuscript draft. GF, GA, LM, AF, MM, MM: final approval of the manuscript. Moreover, the authors declare that the study is original and was not previously presented or published.

## Pre-publication history

The pre-publication history for this paper can be accessed here:

http://www.biomedcentral.com/1472-6963/14/57/prepub
